# M. Trousseau on the Signs of Auscultation in Young Children

**Published:** 1845-02

**Authors:** 


*M. Trousseau on the Signs of Auscultation in young children.*—Every experienced physician must have found—if he has taken the trouble to examine the subject—that auscultation is of comparatively little value in the diagnosis of pulmonic diseases in early life. It is not often that the young patient can be kept sufficiently quiet to enable us to make the proper examination; and, moreover, the respiratory murmur is usually so loud and boisterous—especially upon any excitement—as completely to overpower any abnormal *bruits* that may be present. Fortunately the practitioner does not often feel the need of any extraneous means to aid his diagnosis in the thoracic diseases of infancy; the rational symptoms, as they have been rather absurdly called, being usually quite sufficient. The following remarks were made by M. Trousseau in his clinical lectures at the Hospital Necker.

"If the child be perfectly quiet, the breathing does not sensibly differ from that in the adult; the inspiration is rather noisy, while the expiration is scarcely perceptible; moreover, the former is exceedingly active and slow, while the second, on the contrary, is rapid and purely passive. But, if the child be restless, the inspiration is immediately rapid, and the expansion of the lungs cannot be perceived, while the expiration is, at the same time, slow, and accomplished with the aid of all those muscles which usually concur to the performance of this act. The air issues from the glottis in a small noisy stream. The expiration, therefore, is here essentially active, the very contrary of what it is in the normal state: moreover—and I insist upon this point—it must be very slow, while the act of inspiration is performed rapidly.

"This new rhythm it is very necessary to be aware of, because the character of certain pathological auscultatory sounds, which are thence derived, is more or less altered in consequence. In truth, if, as M. Beau believes—and in this opinion I quite agree with him—the blowing sounds and their numerous modifications really take place and are formed in the larynx, and are transmitted to the ear through the indurated lung by the air in the trachea and bronchi, it must follow that they (the sounds) will be the more distinct and obvious in proportion as the passage of the air is accompanied with the most blowing noise in the larynx. Now this is what we perceive to be the case in the adult. The inspiration is slow, and the expiration is rapid; the inspired air, therefore, passes more silently through the larynx than that which is expired; and thus it is that the blowing sounds are most distinctly audible during the act of expiration. But if—as in the case of the restless child—the inspiration be performed rapidly instead of slowly, the blowing sounds are heard during expiration when it (the child) is calm, and during inspiration when it is restless and crying."—[Med. Chirurg. Rev.

